# Other PHAC publications

**DOI:** 10.24095/hpcdp.44.7/8.09

**Published:** 2024-07

Researchers from the Public Health Agency of Canada also contribute to work published in other journals and books. Look for the following articles published in 2024:

Apelian H, Aho J, Wong E, Cox J. The impact of the COVID-19 pandemic on social determinants of health, mental health, and substance use among key populations affected by sexually transmitted and blood-borne infections in Canada. Can J Public Health. 2024;115(3):432-42. https://doi.org/10.17269/s41997-024-00888-4

Batesole J, Tomkinson GR, Erickson KI, Jurivich D, Lang JJ, McGrath BM, et al. Weakness thresholds are differentially linked to cognitive function by obesity status in older Americans. J Alzheimers Dis Rep. 2024;8(1):601-8. https://doi.org/10.3233/ADR-230190

Bitarafan S, Zhu F, Mirza A, Bernstein CN, Van Domselaar G, Marrie RA, et al. Assessment of dietary intake and its inflammatory potential in persons with pediatric-onset multiple sclerosis. Mult Scler Relat Disord. 2024;86:105599. https://doi.org/10.1016/j.msard.2024.105599

Brazo-Sayavera J, Silva DR, Lang JJ, Tomkinson GR, Agostinis-Sobrinho C, Andersen LB, et al. Physical fitness surveillance and monitoring systems inventory for children and adolescents: a scoping review with a global perspective. Sports Med. Forthcoming 2024. https://doi.org/10.1007/s40279-024-02038-9

Brenner DR, Gillis J, Demers AA, Ellison LF, Billette JM, Zhang SX, et al.; Canadian Cancer Statistics Advisory Committee. Projected estimates of cancer in Canada in 2024. CMAJ. 2024;196(18):E615-23. https://doi.org/10.1503/cmaj.240095

Carter ED, Stewart DE, Rees EE, Bezuidenhoudt JE, Ng V, Lynes S, et al. Surveillance system integration: reporting the results of a global multicountry survey. Public Health. 2024;231:31-8. https://doi.org/10.1016/j.puhe.2024.03.004

Chaput JP, Tomfohr-Madsen L, Carney CE, Robillard R, Sampasa-Kanyinga H, Lang JJ. Examining sleep characteristics in Canada through a diversity and equity lens. Sleep Health. 2024;10(3):316-20. https://doi.org/10.1016/j.sleh.2024.02.001

De Rubeis V, Griffith LE, Duncan L, Jiang Y, de Groh M, Anderson LN. Self-reported chronic conditions and COVID-19 public health measures among Canadian adults: an analysis of the Canadian Longitudinal Study on Aging. Public Health. 2024;231:99-107. https://doi.org/10.1016/j.puhe.2024.03.015

Garritty C, Nussbaumer-Streit B, Hamel C, Devane D. Rapid reviews methods series: assessing the appropriateness of conducting a rapid review. BMJ Evid Based Med. Forthcoming 2024. https://doi.org/10.1136/bmjebm-2023-112722

Gholi Zadeh Kharrat F, Gagne C, Lesage A, Garipy G, Pelletier JF, Brousseau-Paradis C, et al. Explainable artificial intelligence models for predicting risk of suicide using health administrative data in Quebec. PLoS One. 2024;19(4):e0301117. https://doi.org/10.1371/journal.pone.0301117

Lang JJ, Prince SA, Merucci K, Cadenas-Sanchez C, Chaput JP, Fraser BJ, et al. Cardiorespiratory fitness is a strong and consistent predictor of morbidity and mortality among adults: an overview of meta-analyses representing over 20.9 million observations from 199 unique cohort studies. Br J Sports Med. 2024;58(10):556-66. https://doi.org/10.1097/PHH.0000000000001884

Liu L, Pollock NJ, Contreras G, Xu Y, Thompson W. Hospitalizations and emergency department visits for self-harm in Canada during the first two years of the COVID-19 pandemic: a time series analysis. J Affect Disord. 2024;355:505-12. https://doi.org/10.1016/j.jad.2024.03.123

MacNeil A, Cottagiri SA, Villeneuve PJ, Jiang Y, de Groh M, Fuller-Thomson E. Incident functional limitations among older adults with diabetes during the COVID-19 pandemic: an analysis of prospective data from the Canadian Longitudinal Study on Aging. Can J Diabetes. 2024:S1499-2671(24)00057-1. https://doi.org/10.1016/j.jcjd.2024.02.005

Nelson CRM, Dzakpasu S, Moore AM, Darling EK, Edwards W, Murphy P, et al. Diabetes mellitus in pregnancy across Canada. BMC Pregnancy Childbirth. 2024;24(1):349. https://doi.org/10.1186/s12884-024-06534-8

Pillay J, Rahman S, Klarenbach S, Reynolds DL, Tessier LA, Thriault G, […] Garritty C, Traversy G, et al. Screening for lung cancer with computed tomography: protocol for systematic reviews for the Canadian Task Force on Preventive Health Care. Syst Rev. 2024;13(1):88. https://doi.org/10.1186/s13643-024-02506-3

Ricci C, Subburaj D, Lim K, Shukla N, Kaur J, Xie L, Laverty M, Zakaria D, et al. Second malignant neoplasms within 5 years from first primary diagnosis in pediatric oncology patients in Canada: a population-based retrospective cohort study. Front Oncol. 2024;14:1376652. https://doi.org/10.3389/fonc.2024.1376652

Scott MM, Mnard A, Sun AH, Murmann M, Ramzy A, Rasaputra P, et al. Building evidence to advance health equity: a systematic review on care-related outcomes for older, minoritised populations in long-term care homes. Age Ageing. 2024;53(4):afae059. https://doi.org/10.1093/ageing/afae059

Shephard R, Uy J, Otterman V, Betker C, Sandhu HS, Tjaden L, […] Payne E, Fang L. The Core Competencies for Public Health in Canada: opportunities and recommendations for modernization. J Public Health Manag Pract. 2024;30(3):432-41. https://doi.org/10.1097/PHH.0000000000001884

Vanhelst J, Lang JJ, Matelot D, Carr F, Mercier D, Ulmer Z, et al. Cardiorespiratory fitness has declined among French children since 1999, although the decline appears to be getting smaller. Scand J Med Sci Sports. 2024;34(5):e14641. https://doi.org/10.1111/sms.14641

Weiler HA, Rana H, McCrea J, Loukine L, Bonvalot Y, Nguyen L, […] Vercammen K, Luo W, Nicholson C, et al. Adherence to vitamin D supplementation recommendations for breastfed infants and young children: an analysis of Canadian Community Health Survey data cycles from 2015 to 2018. J Nutr. 2024;154(5):1665-75. https://doi.org/10.1016/j.tjnut.2024.03.016

Zahan R, Osgood ND, Plouffe R, Orpana H. A dynamic model of opioid overdose deaths in Canada during the co-occurring opioid overdose crisis and COVID-19 pandemic. Int J Environ Res Public Health. 2024;21(4):442. https://doi.org/10.3390/ijerph21040442

